# Mapping Intracellular Volume Fraction With Susceptibility Source Decomposition as a Marker for Tissue Cellularity

**DOI:** 10.1002/jmri.70194

**Published:** 2025-12-10

**Authors:** Giulia Debiasi, Oliver C. Kiersnowski, Giovanni Librizzi, Luca Roccatagliata, Renzo Manara, Mauro Costagli, Alessandra Bertoldo, Chunlei Liu

**Affiliations:** ^1^ Department of Electrical Engineering and Computer Sciences University of California Berkeley California USA; ^2^ IRCCS Ospedale Policlinico San Martino Genova Italy; ^3^ Padova Neuroscience Center, University of Padova Padova Italy; ^4^ Neuroradiology, Department of Neurosciences University of Padova Padova Italy; ^5^ Department of Health Sciences University of Genova Genova Italy; ^6^ Department of Medicine University of Padova Padova Italy; ^7^ Department of Neuroscience, Rehabilitation, Ophthalmology, Genetics, Maternal and Child Health University of Genova Genova Italy; ^8^ Department of Information Engineering University of Padova Padova Italy; ^9^ Helen Wills Neuroscience Institute, University of California Berkeley California USA

**Keywords:** cellularity, diffusion, glioblastoma, magnetic susceptibility

## Abstract

**Background:**

Pathophysiological changes affect tissue cell composition and density. For example, neurodegenerative disorders and brain tumors are associated with cell loss and abnormal accumulation, respectively. In these scenarios, if monitored and tracked, tissue cellularity might be used to inform clinical diagnosis and management.

**Purpose:**

To propose and evaluate a new marker of tissue cellularity, called susceptibility‐Derived Cellularity Index (χDCI), that would be readily available for clinical applications with fast acquisition and at high resolution.

**Study Type:**

Retrospective study.

**Population/Subjects:**

24 healthy subjects (7/17 M/F, 70 ± 11 years) and 21 patients with IDH‐wild type glioblastoma (16/5 M/F, 65 ± 8 years).

**Field Strength/Sequence:**

3 T MRI sequences including 3D T1w pre‐ and post‐contrast agent injection, 3D T2w, 3D FLAIR, 3D multi‐echo gradient recalled echo, 2D diffusion weighted imaging.

**Assessment:**

χDCI was computed based on parameters estimated with DECOMPOSE‐QSM. The Neurite Density Index (NDI) was estimated with the NODDI model. T1w images were used for region of interest (ROIs) segmentations with FreeSurfer (i.e., cortical gray matter, white matter, thalamus, caudate, putamen, pallidum, hippocampus and amygdala). For the patients with glioblastoma, regions of contrast enhancement, necrosis, and edema were also included in the analysis.

**Statistical Tests:**

Pearson's correlation analysis between mean χDCI and NDI values in the ROIs was carried out separately for the two cohorts of participants (significance level = 0.05, after correction for multiple comparisons).

**Results:**

Significant correlations were observed between χDCI and NDI in white matter (*r* = 0.56) and putamen (*r* = 0.69) for the healthy participants. Significant positive correlations were also found in white matter (*r* = 0.6), pallidum (*r* = 0.48), putamen (*r* = 0.79), thalamus (*r* = 0.64) and edema (*r* = 0.69) for the patient cohort.

**Data Conclusion:**

χDCI is proposed as a marker of tissue cellularity. The significant associations between χDCI and NDI in several regions investigated in the present study support the potential of χDCI as a proxy of intracellular volume fraction.

**Level of Evidence:**

3.

**Technical Efficacy:**

Stage 1.

## Plain Language Summary

Tissue cellularity is affected by pathophysiological changes and imaging markers of cellular components could aid clinical management of brain diseases. The authors evaluated a potential new marker of tissue cellularity, called susceptibility‐Derived Cellularity Index (χDCI), that is computed based on the decomposition of magnetic susceptibility sources. χDCI was compared to the neurite density index, derived from diffusion MRI, in healthy and lesioned brain regions. The observed significant correlations suggest χDCI as a potential marker of intracellular volume fraction. These findings support the use of χDCI to track and monitor cellular pathological brain changes. χDCI could be readily employed for clinical applications.

## 1 Introduction

Pathological brain changes often affect the density of the cellular components. For example, neuronal death is the ultimate consequence of the accumulation of amyloid‐β (Aβ) and hyperphosphorylation of tau protein in Alzheimer's disease [1]; selective loss of dopaminergic neurons in the substantia nigra characterizes Parkinson's disease [2]; demyelination and progressive axonal degeneration are the pathological features of multiple sclerosis [3]; cell loss is also observed in amyotrophic lateral sclerosis [4]; and cell proliferation is a hallmark of brain tumors [5]. Histopathological analysis is usually performed on tissue samples extracted during surgery, e.g., for brain tumors, or occasionally on post‐mortem tissues for other neurodegenerative diseases. Non‐invasive detection of certain cells in the brain can be performed with positron emission tomography and optical imaging. However, low specificity and sensitivity, low spatial resolution and poor tissue penetrance are among the limitations associated with these techniques [6].

MRI is commonly employed to study tissue microstructure. To date, some of the most widely used MRI techniques for microstructure imaging are diffusion weighted imaging and related techniques such as diffusion tensor imaging (DTI) [7] and higher order tensor diffusion including diffusion kurtosis imaging [8, 9, 10], which are sensitive to the random motion of water molecules in biological tissues. Particularly, the neurite orientation dispersion and density imaging (NODDI) model [11], which separates the diffusion signal into three compartments, namely intracellular, extracellular and cerebrospinal fluid (CSF) compartments, has been applied to assess microstructure and cellularity in the brain [12]. The NODDI model estimates parametric maps of neurite density index (NDI), orientation dispersion index (ODI) and isotropic volume fraction (ISO). NDI has been used in several clinical applications. For example, it has been previously shown that NDI is sensitive to cortical and subcortical pathological changes of Alzheimer's disease [13, 14]. In amyotrophic lateral sclerosis, reduced NDI was found throughout the corticospinal tract and across the corpus callosum [15]. Reduced NDI was also observed in the white matter of patients with multiple sclerosis compared to controls [16, 17]. In patients with Parkinson's disease, abnormalities in NDI have been reported compared with controls [18, 19]. Furthermore, increased NDI has also been observed in the contrast‐enhancing region of gliomas [20, 21], whereas edema showed reduced NDI compared to the apparently healthy white matter [22]. These findings potentially reflect the complex tissue composition of the tumor, in which aggregates of densely packed tumor cells in the contrast‐enhancing region result in increased NDI. On the other hand, vasogenic edema increases the isotropic volume fraction, consequently reducing NDI. For patients with glioblastoma, NDI was also proposed for differential diagnosis [23] and for differentiating between non‐enhancing tumor and vasogenic edema [24].

The recently developed DECOMPOSE‐QSM algorithm [25] for separating sub‐voxel magnetic susceptibility sources provides a new tool to study tissue microstructure. The method relies on a gradient‐recalled (GRE) multi‐echo sequence that is readily available on clinical scanners and provides high spatial resolution. DECOMPOSE‐QSM models the signal originating from three magnetic susceptibility compartments and estimates their volume fractions. The aim of the present study is to propose a susceptibility‐Derived Cellularity Index, called χDCI, as a marker of tissue cellularity, based on the parameters estimated from DECOMPOSE‐QSM. To evaluate the proposed χDCI, a comparison with NDI across some brain regions is performed.

## 2 Materials and Methods

### 2.1 Subjects

A cohort of 24 healthy volunteers (7/17 M/F, 70 ± 11 years) and a cohort of 21 patients (16/5 M/F, 65 ± 8 years) were retrospectively included in this study. Inclusion criteria for the healthy volunteers were: (i) no neurological and psychiatric diseases; (ii) multi‐echo GRE and diffusion MRI sequences acquired. For the patient cohort inclusion criteria included: (i) newly diagnosed IDH‐wild type glioblastoma; (ii) multi‐echo GRE and diffusion MRI scans acquired before surgical resection. All subjects provided informed, written consent in accordance with the study protocols (n. AOP2971 and N. Registro CER Liguria: 168/2022 ‐ DB id 12248) approved by the Institutional Review Boards.

### 2.2 MRI Acquisition

Healthy subjects were acquired on a 3 T Siemens Magnetom Prisma scanner using a 64‐channel head–neck coil, with the following MRI protocol: 3D T1‐weighted (T1w), 3D Fluid Attenuation Inversion Recovery (FLAIR), 3D multi‐echo GRE sequence and diffusion weighted images. Details on the sequence parameters can be found in Table 1.

**TABLE 1:** Sequences parameters for the healthy cohort.

	TE (ms)	TR (ms)	Voxel size (mm^3^)	FOV (mm^2^)	Number of slices	Acquisition time (min)	Additional details
T1w	2.98	2300	1 × 1 × 1	256 × 256	176	5:30	Inversion time = 900 ms
FLAIR	383	4500	1 × 1 × 1	256 × 256	176	5:53	
Multi‐echo GRE	TE_1_/ΔTE = 5.6/5.6	51	1 × 1 × 1	224 × 224	144	8:45	Eight echoes Flip angle = 18**°**
Diffusion weighted imaging	85	4000	2.5 × 2.5 × 2.5	240 × 240	60	4:48	#gradient directions, *b* value (s/mm^2^): 7, *b* = 0 30, *b* = 1000 30, *b* = 2000 Multiband factor = 2

Patient data were acquired with a 3 T Philips Ingenia scanner using a 32‐channel head coil. The acquisition protocol for this cohort consisted of the following: 3D T1w pre‐ and post‐gadolinium injection (T1w‐Gd), 3D T2‐weighted (T2w), 3D FLAIR, 3D multi‐echo GRE sequence and diffusion‐weighted images. Details on sequence parameters can be found in Table 2. Adaptive coil combination was used for both scanners.

**TABLE 2:** Sequences parameters for the patient cohort.

	TE (ms)	TR (ms)	Voxel size (mm^3^)	FOV (mm^2^)	Number of slices	Acquisition time (min)	Additional details
T1w (pre‐ and post‐contrast agent injection)	3	6.7	1 × 1 × 1	240 × 240	181	3:14	
T2w	280	3000	1 × 0.94 × 0.94	256 × 256	181	4:42	
FLAIR	360	8000	1.12 × 1.12 × 1.12	221 × 221	326	5:36	Reconstructed to 0.56 × 0.62 × 0.62 mm^3^ FOV = 400 × 400 mm^2^
Multi‐echo GRE	TE_1_/ΔTE = 5/5	44	1 × 1 × 1	240 × 240	181	7:00	Eight echoes Flip angle = 25**°**
Diffusion weighted imaging	104	3700	2 × 2 × 2	112 × 112	78	7:18	#gradient directions, *b* value (s/mm^2^): 12, *b* = 0 8, *b* = 300 32, *b* = 1000 64, *b* = 2000 Multiband factor = 3

### 2.3 Structural Images Processing

Structural images (i.e., T1w, T2w, FLAIR, T1w‐Gd) were bias field corrected [26] and brain extracted [27]. Then, T2w, FLAIR and T1w‐Gd were registered to the native T1w space with Advanced Normalization Tools (ANTs) [28]. For each participant, FreeSurfer [29] was used on the T1w image to generate cortical gray matter (GM), white matter (WM) and subcortical regions masks. A linear registration was estimated with ANTs from the T1w space to the first echo magnitude image of the 3D GRE sequence. The estimated transformations were applied to cortical GM, WM and subcortical structures masks. Additionally, a non‐linear transformation was estimated from the T1w space to the symmetric T1w in Montreal Neurological Institute (MNI) space (https://www.bic.mni.mcgill.ca/ServicesAtlases/ICBM152NLin2009) with ANTs.

### 2.4 Tumor Segmentation

Tumor masks were obtained with an automatic tool [30, 31] which requires as input the four structural images. Three tissue types were segmented, namely edema/non‐enhancing tumor, necrosis and contrast‐enhancing tumor. Segmentations were then inspected and refined by neuroradiologists with experience in the glioma field. The tumor masks were then warped to the susceptibility space and the diffusion space with the previously estimated transformations.

### 2.5 Diffusion Images Processing and Quantification

MRtrix [32] was used to carry out the preprocessing which included denoising and correction for B_0_ inhomogeneities, eddy currents and motion [33, 34], after discarding volumes affected by interslice instabilities [35]. A linear transformation was estimated with ANTs between the average *b* = 0 image and the T2w image and then applied to the masks of interest for the analysis in the diffusion space. The NODDI model was fitted with a publicly available toolbox (http://mig.cs.ucl.ac.uk) resulting in NDI, ISO and ODI maps for each participant.

### 2.6 Magnetic Susceptibility Processing and Definition of χDCI

**FIGURE 1:**
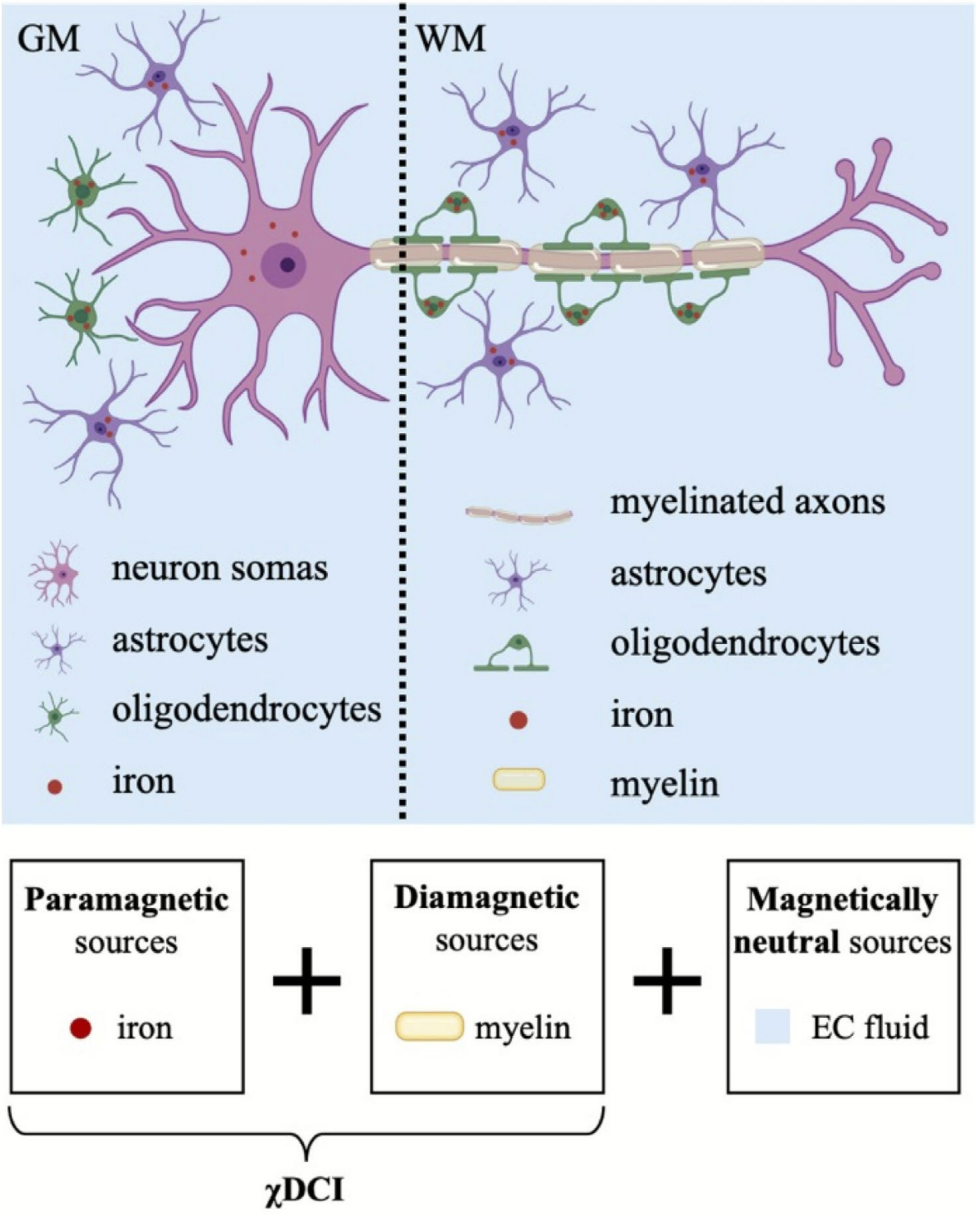
Graphical representation of tissue microstructure based on which χDCI is defined. Sources of magnetic susceptibility in gray matter (GM) and white matter (WM) are depicted, and an incomplete list of potential cellular/molecular contributors is shown. Paramagnetic, diamagnetic and magnetically neutral sources are mainly represented by iron, myelin and extracellular (EC) fluid, respectively. χDCI accounts for the paramagnetic and diamagnetic components. Created in BioRender. Debiasi, G. (2025) https://BioRender.com/r7oz93p; modified in PowerPoint.

Whole brain magnitude and unfiltered phase images were used to quantify magnetic susceptibility and DECOMPOSE‐QSM parameters. The first echo magnitude image of the GRE acquisition was used to perform brain extraction with the Brain Extraction Tool (BET) of FSL [36]. Magnetic susceptibility maps were quantified with STISuite (https://chunleiliulab.github.io/software/#STI) for each echo as follows. First, Laplacian phase unwrapping [37] was achieved and then the brain mask was eroded by 3 mm to account for signal drops at the boundaries of the brain. For the patients with glioblastoma, the binarized tumor mask was added back to the eroded brain mask to avoid loss of information in the case of cortical tumors. Background field removal and dipole inversion steps were carried out with V‐SHARP [38, 39] and iLSQR [40] algorithms, respectively. For the patient cohort, STAR‐QSM [41] was used as an additional method for dipole inversion for comparison. Magnitude and QSM map of each echo were given as input to the DECOMPOSE‐QSM model. For the healthy cohort only the first seven echoes were used to match the TEs range of the patient data. According to the assumptions of the DECOMPOSE‐QSM model [25], C_+_ + C_−_ + C_0_ = 1, with C_+_, C_−_ and C_0_ being the volume fractions of the paramagnetic, diamagnetic and magnetically neutral compartments, respectively. Therefore, in addition to the estimation of paramagnetic and diamagnetic component susceptibilities (PCS and DCS, respectively), DECOMPOSE‐QSM also estimates the volume fraction of the three compartments. With C_0_ representing the extracellular fluid, by computing 1‐C_0_, a measure of the intracellular volume fraction in each voxel is therefore obtained. This measure is referred to as susceptibility‐Derived Cellularity Index (χDCI). A simplified graphical representation of the biological context in which χDCI is defined is given in Figure 1. Gray matter is mostly made up of neuronal somas and glial cells, while in the white matter, an abundance of myelinated axons, oligodendrocytes and astrocytes can be found. Oligodendrocytes are the most common type of glial cell, and their density increases from deep cortical layers up to white matter [42]. The surrounding space is filled with extracellular fluid. Other types of cells such as microglial cells and blood‐vessel‐related cells are present as well. Each voxel of the brain as captured by GRE MRI can be described as the superposition of three compartments with distinct magnetic susceptibility. The three magnetic susceptibility sources are paramagnetic, diamagnetic and magnetically “neutral”, originating from iron, myelin and the extracellular fluid, respectively. Each of these sources is accounted for by estimating their volume fractions (i.e., C_+_, C_−_, C_0_).

### 2.7 Statistical Analysis

The regions of interest (ROIs) selected for the analysis were cortical GM, WM, thalamus, caudate, putamen, pallidum, hippocampus, and amygdala. All regions except cortical GM were eroded by one voxel in both the χDCI and NDI native spaces to minimize partial volume effects. The substantia nigra and red nuclei were excluded from the analysis because they occupy a very limited number of voxels in diffusion weighted images. Their volume is less than 300 mm^3^ [43] and the volume of a voxel in diffusion space is approximately 16 mm^3^. This results in only a small number of voxels available for analysis (e.g., 18 voxels), and after erosion only few or none would remain. For the healthy subjects, the average value of each ROI for the map of interest was computed from both hemispheres, while for the patient cohort only the hemisphere contralateral to the tumor was considered. Pearson's correlation was first computed between the χDCI and NDI means of all pooled ROIs across the two cohorts. Correlation coefficients were then computed for each individual ROI. In the patient cohort, the analysis was also extended to the edema/non‐enhancing, necrotic, and contrast‐enhancing tumor components. Since it is known that different QSM algorithms generate different magnetic susceptibility values and that QSM has not been investigated widely in glioblastomas, correlation and Bland–Altman analysis were also conducted on STAR‐QSM‐derived and iLSQR‐derived χDCI maps for the patient cohort. Furthermore, to assess reproducibility, relationships between ROI means of χDCI and NDI of the two groups were investigated with correlation and Bland–Altman analysis. The significance level was set to 0.05 for all the analysis, if not otherwise stated. Correction for multiple comparisons was applied by means of the Benjamini‐Hochberg procedure. The statistical analysis was carried out in Matlab R2024b (for Bland–Altman analysis: BlandAltmanPlot (https://github.com/thrynae/BlandAltmanPlot/releases/tag/v1.2.1), GitHub.).

## 3 Results

### 3.1 The Proposed χDCI as A Marker of Tissue Cellularity

**FIGURE 2:**
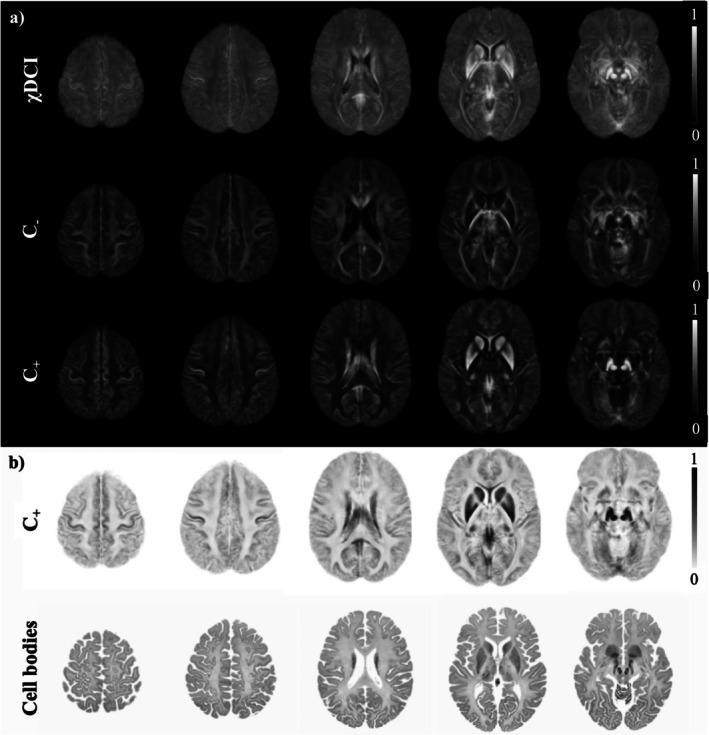
Group averaged maps in MNI space. (a) χDCI, C_−_ and C_+_ maps. Anatomical locations visible on χDCI are even more highlighted when C_−_ and C_+_ are considered separately. (b) C_+_ map and BigBrain. The BigBrain was derived from a single postmortem individual. Iron‐rich cortical areas and the deep gray matter structures shown on C_+_ are also visible on the BigBrain. The colormap for C_+_ has been reversed to visually match the cell body staining (Merker stain) of the BigBrain.

Figure 2 shows the average maps of χDCI, C_−_ and C_+_ computed in MNI space for healthy subjects (5/7 M/F, 68 ± 13 years). Higher χDCI values were observed in the cortical gray matter compared to white matter, with elevated values also evident in deep gray matter structures. Cerebrospinal fluid (CSF) regions appear dark with values close to zero, while bright χDCI voxels are seen in the ventricles due to averaging of the choroid plexus among the subjects in MNI space. The C_−_ map shows highly myelinated structures such as optic radiation and internal and external capsules. The C_+_ map highlights iron‐rich structures such as caudate, putamen, pallidum, red nuclei, and substantia nigra, as well as cortical areas such as the precentral gyrus.

When the colormap is inverted for C_+_, it presents a high contrast similarity with the BigBrain [44] (Figure 2b), particularly in deep gray matter structures and cortical layers.

### 3.2 Qualitative Comparison of χDCI and NDI Maps

**FIGURE 3:**
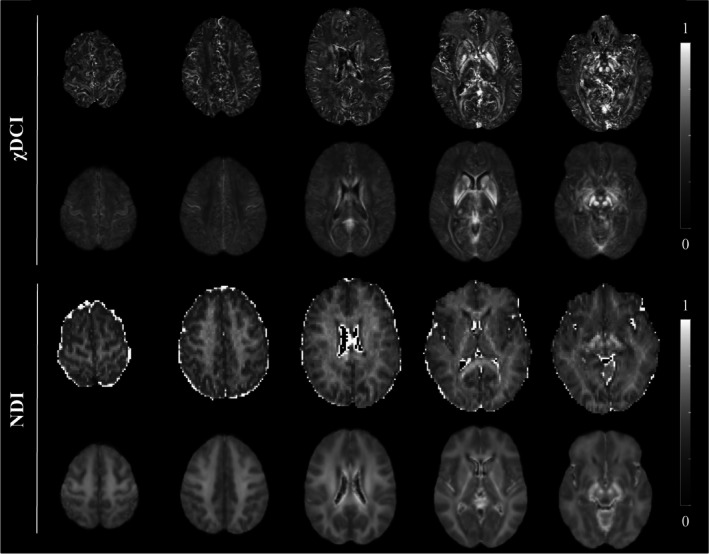
Comparison of χDCI and NDI maps. For both maps, the first row shows examples of axial slices of a representative subject and the second row shows the group averaged map computed in MNI space. The hyperintense NDI artifact in cerebrospinal fluid has been masked out in the averaged map.

Examples of χDCI and NDI maps of a healthy participant are reported in Figure 3 (first and third row, respectively), together with average maps computed for each contrast in MNI space (Figure 3, second and fourth row, respectively). In general NDI highlights white matter and more closely resembles the C_−_ map (Figure 2). On the other hand, χDCI shows a different contrast between gray and white matter, delineating cortical areas as well as deep gray matter structures. Boundaries of the deep gray nuclei are more defined in χDCI with respect to NDI, both at the single‐subject and average level. Moreover, the common NDI artifact related to the CSF is not present in χDCI. In the pathological cohort, the same observations can be made. As can be observed from Figure S1, 1‐ISO does not present specific tissue contrast but clearly shows the CSF voxels characterized by isotropic diffusion.

**FIGURE 4:**
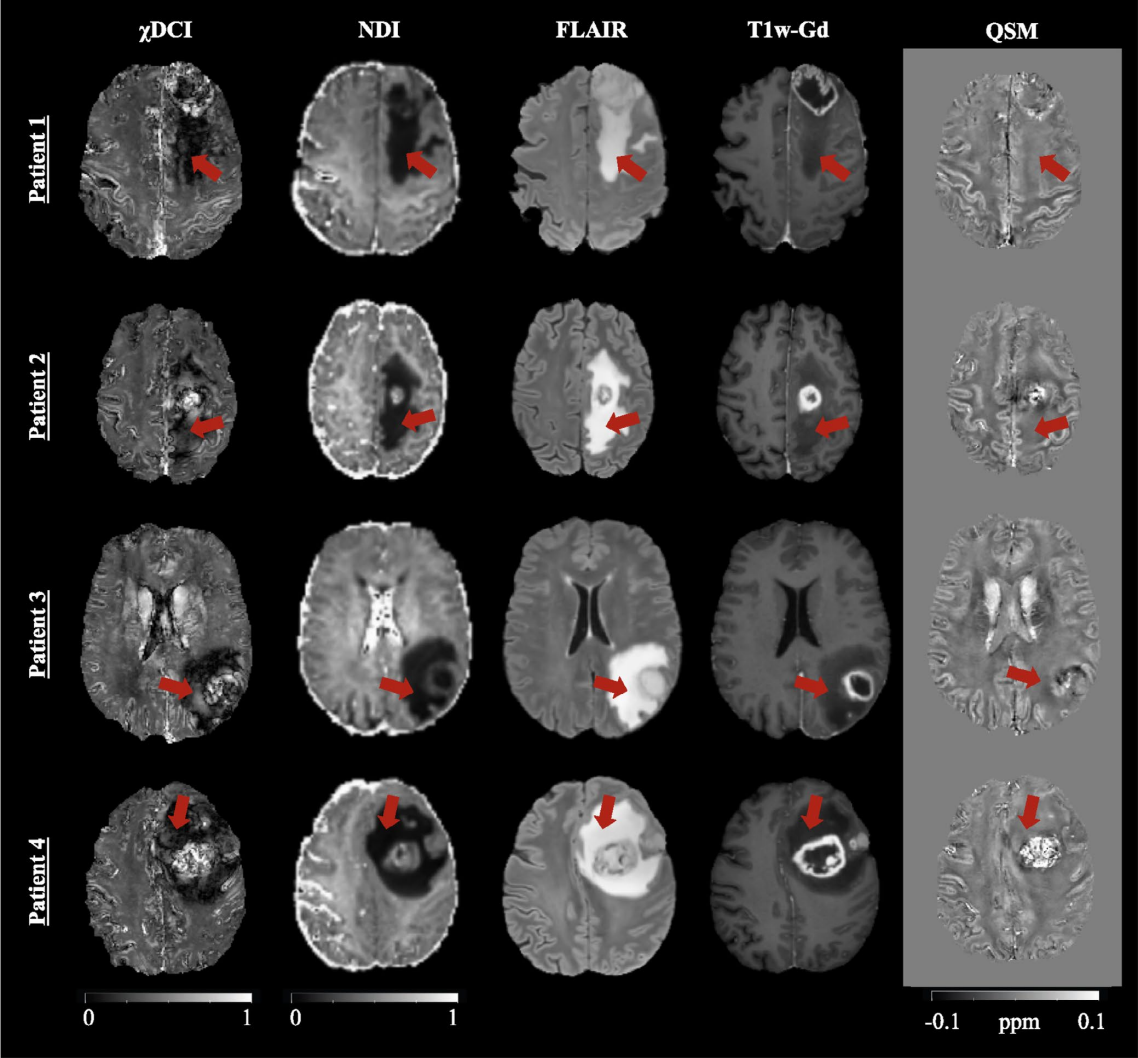
Examples of images of four patients with glioblastoma. From left to right: χDCI, NDI, FLAIR, T1w‐Gd and QSM. Heterogeneity within the different tumor regions (i.e., contrast enhancement, necrosis and edema) can be appreciated on χDCI but not on NDI. For visualization purposes, all images are warped to the χDCI native space. Red arrows point at a location in which the edema is heterogeneous on χDCI.

Figure 4 presents maps of four representative patients with glioblastoma. On NDI, T2‐FLAIR, T1w‐Gd and QSM, edema appears as a generally homogeneous area. In comparison, χDCI shows a complex heterogeneity (as indicated by the red arrows, Figure 4). Taken together, these results highlight some potential advantages of χDCI over NDI as a marker for tissue cellularity in the brain.

### 3.3 χDCI and NDI Association Has Regional Variations

A significant positive correlation was found between χDCI and NDI mean values for ROIs of the healthy participants (*r* = 0.63) and the patient with glioblastoma (*r* = 0.81). Scatterplots of these correlations are reported in Figure S2.

**FIGURE 5:**
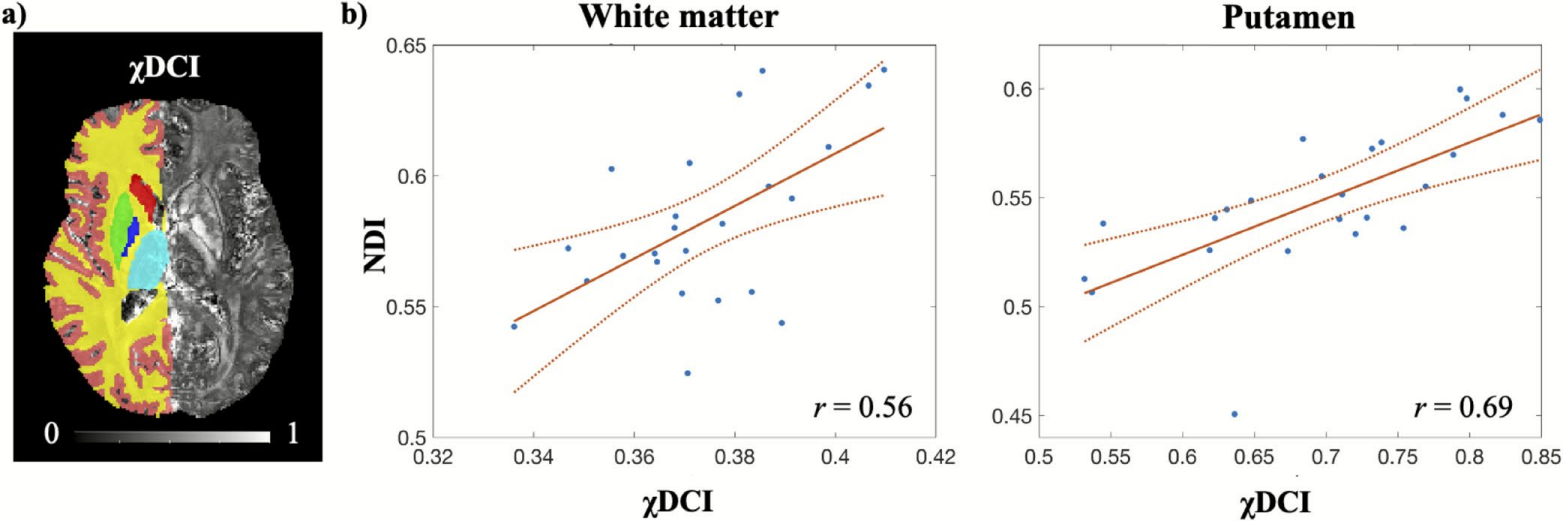
Positive associations between χDCI and NDI in healthy participants. (a) ROIs segmentation of a healthy subject. Only the segmentations for one hemisphere are reported as examples on the left side of the image, and they are overlaid on χDCI. ROIs are shown with different colors (cortical GM—pink, WM—yellow, caudate—red, putamen—green, pallidum—blue, thalamus—light blue). (b) Significant associations between χDCI and NDI in the investigated ROIs for the healthy cohort. *r* indicates the correlation coefficient. Data points are reported as “.”, the fit is represented by the continuous line and the confidence bounds are indicated by the dotted lines.

From the correlation analysis at the ROI‐level for the healthy cohort, significant positive associations were observed in white matter (*r* = 0.56) and putamen (*r* = 0.69), as reported in Figure 5b. No significant correlation was instead found for cortical GM (*p* = 0.2, *p*: *p*‐value after correction for multiple comparisons), amygdala (*p* = 0.3), hippocampus (*p* = 0.9), pallidum (*p* = 0.4), thalamus (*p* = 0.3) and caudate (*p* = 0.3). A subset of the ROIs considered for the analysis is shown in Figure 5a.

**FIGURE 6:**
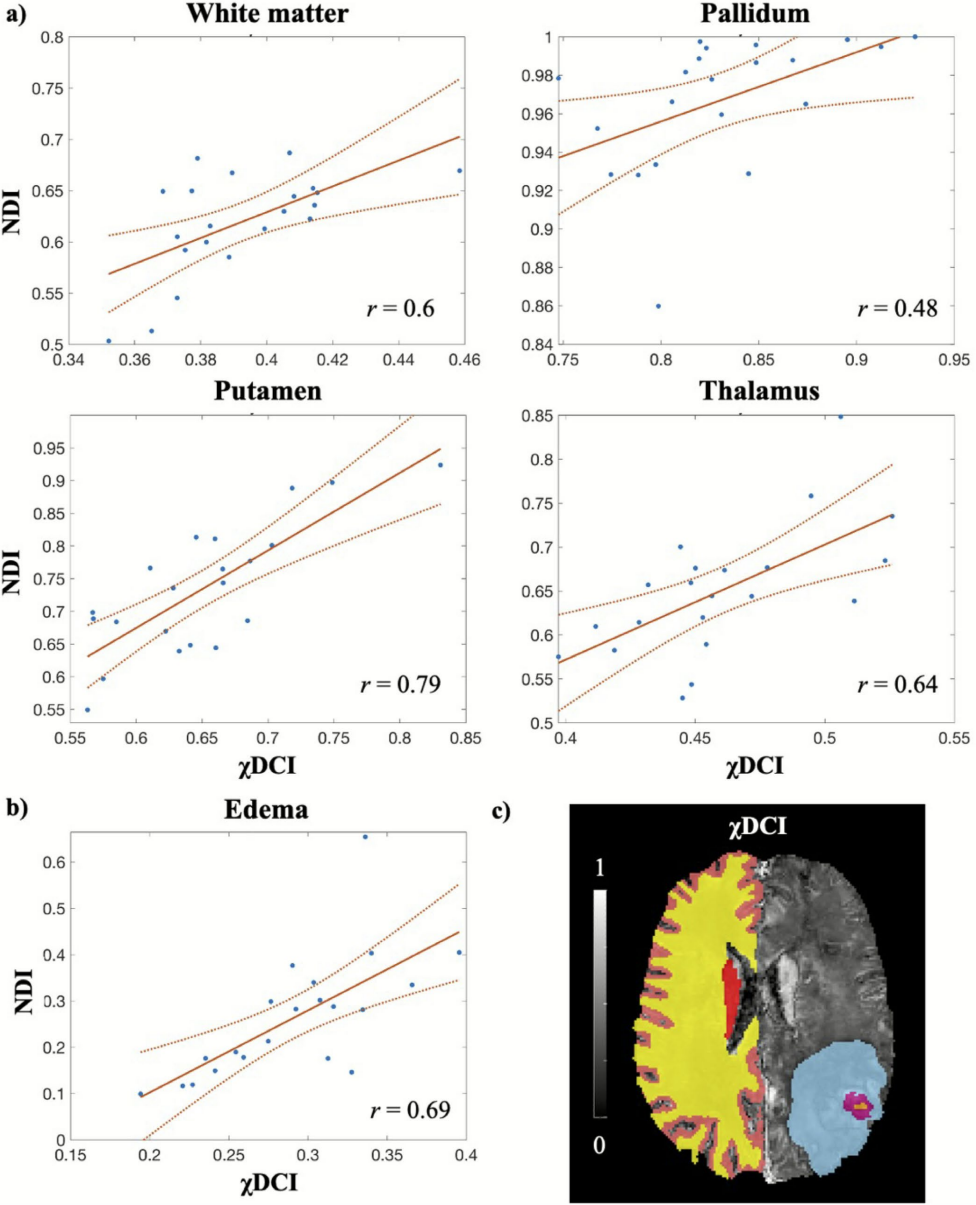
Positive associations between χDCI and NDI in the patient cohort. (a) Significant correlations between χDCI and NDI in the investigated healthy ROIs for the patient cohort. (b) Significant correlations between χDCI and NDI in the investigated tumor ROIs for the patient cohort. *r* indicates the correlation coefficient. Data points are reported as “.”, the fit is represented by the continuous line and the confidence bounds are indicated by the dotted lines. Results here reported refer to χDCI estimated with iLSQR‐derived susceptibility maps. (c) ROIs segmentation for a patient with glioblastoma. Only the segmentations in the hemisphere contralateral to the tumor are considered. ROIs are shown with different colors (cortical GM—pink, WM—yellow, caudate—red, edema—light blue, contrast enhancement—magenta, necrosis—orange) and they are overlaid on χDCI.

Positive associations were also found between χDCI and NDI for the patients with glioblastoma and scatterplots of the correlations are reported in Figure 6. Considering the apparently healthy tissues (Figure 6a), significant correlations were observed for white matter (*r* = 0.6), pallidum (*r* = 0.48), putamen (*r* = 0.79) and thalamus (*r* = 0.64). No association was found for cortical GM (*p* = 0.2), amygdala (*p* = 0.7), hippocampus (*p* = 0.5) and caudate (*p* = 0.7). No significant association was found for necrosis (*p* = 0.2) and contrast enhancement (*p* = 0.7) areas, while a significant positive association was observed for edema (*r* = 0.69). The corresponding scatterplot is reported in Figure 6b. An example of segmented ROIs for the patient with glioblastoma is reported in Figure 6c.

### 3.4 Reproducibility of χDCI in the Patient Cohort

**FIGURE 7:**
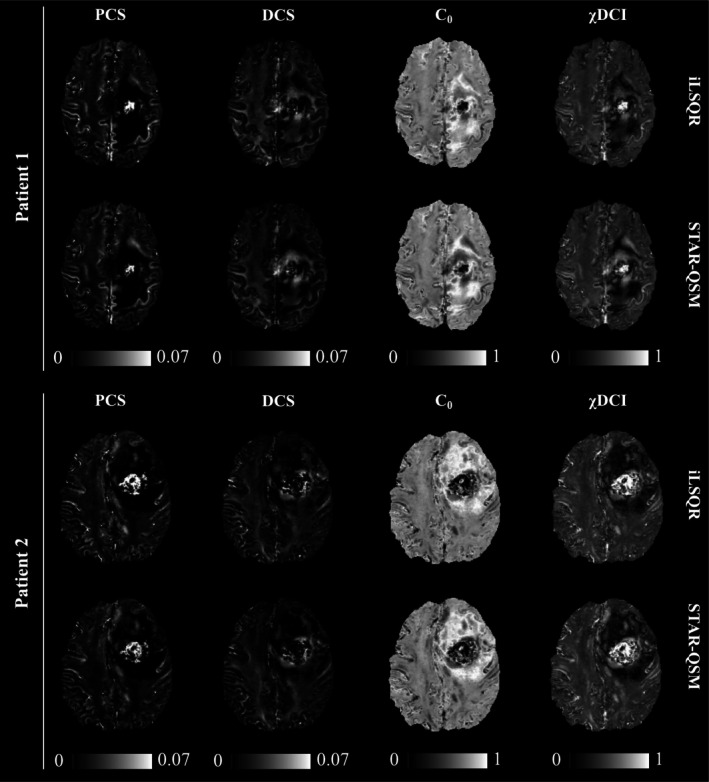
Estimated parameters of DECOMPOSE‐QSM for two representative patients with glioblastoma. Top and bottom rows for each patient show maps derived from iLSQR and STAR‐QSM, respectively. Each column, from left to right, represents the Paramagnetic Component Susceptibility (PCS), the Diamagnetic Component Susceptibility (DCS), C_0_ and χDCI. DCS is shown as absolute value.

Figure 7 presents two representative patients for which DECOMPOSE‐QSM has been applied on magnetic susceptibility maps derived from both iLSQR and STAR‐QSM. Each column reports a different parameter estimate of the model and the proposed χDCI. The maps derived from the two dipole inversion algorithms visually appear comparable, and in fact, the QSM maps used as input for DECOMPOSE‐QSM only present small variations that have been previously assessed to be less than 2% [41]. Repeating the correlation analysis between χDCI (based on STAR‐QSM) and NDI, significant positive associations were found for white matter (*r* = 0.56), pallidum (*r* = 0.54), putamen (*r* = 0.81) and thalamus (*r* = 0.69), but not for cortical GM (*p* = 0.1), amygdala (*p* = 0.7), hippocampus (*p* = 0.6) and, caudate (*p* = 0.7). Scatterplots of the results are reported in Figure S3. In the glioblastoma tissues, a significant positive correlation was observed for edema (*r* = 0.78), but not for necrosis (*p* = 0.1) and contrast enhancement (*p* = 0.7). For completeness, the correlation coefficients computed by using χDCI derived with the two different dipole inversion methods are reported in Table S1. Bland–Altman analysis revealed a significant but small bias between STAR‐QSM‐ and iLSQR‐derived χDCI values for both the apparently healthy (*b* = −0.0038; *b*: mean difference) and the tumor tissues (*b* = 0.011). However, no specific trend is present within the limits of agreement as can be observed in Figure S4.

**FIGURE 8:**
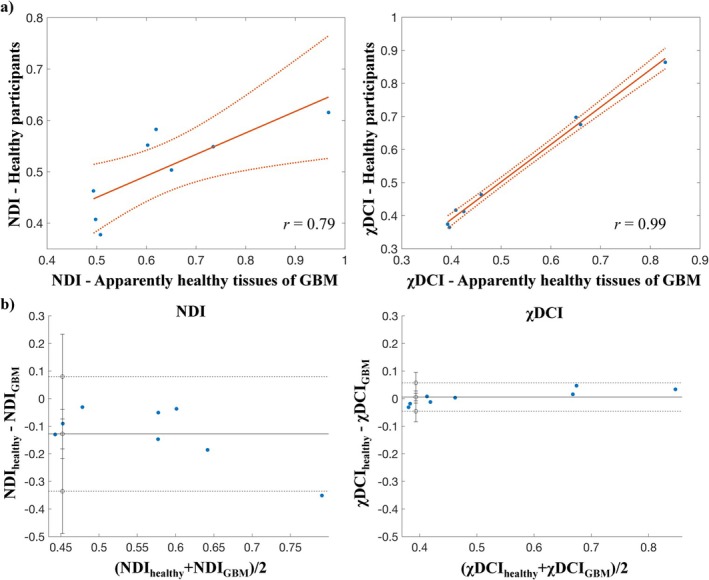
Reproducibility of χDCI and NDI across ROIs values of the two population cohorts. (Panel a) Scatterplots of NDI and χDCI. Values of ROIs from the healthy participants are reported on the y‐axis, while ROIs belonging to the apparently healthy tissues of the patients with glioblastoma (GBM) are on the x‐axis. *r* indicates the correlation coefficient. Data points are reported as “.”, the fit is represented by the continuous line and the confidence bounds are indicated by the dotted lines. (Panel b) Bland–Altman plots for NDI and χDCI. Continuous and dashed lines represent the mean difference and the limits of agreement, respectively. Error bars indicate the 95% confidence intervals.

A significant correlation was found for χDCI (*r* = 0.99) and NDI (*r* = 0.79) and corresponding scatterplots for the data are reported in Figure 8 (panel a), together with the Bland–Altman plots (panel b). A negligible bias was observed for χDCI (*b* = 0.0057, *p* = 0.56; *p*: *p*‐value). Instead, a negative bias was observed for NDI (*b* = −0.13), even though all data points except one are found within the limits of agreement.

## 4 Discussion

This study introduced χDCI, a novel marker of tissue cellularity derived from magnetic susceptibility compartmentalization of brain tissues. The findings showed that χDCI represents the intracellular volume fraction of a voxel and showed positive associations with the widely used NDI from the diffusion‐based NODDI model in several ROIs, including white matter, across both healthy and patient cohorts. However, most gray matter ROIs did not show significant associations. Together, the data suggest that χDCI and NDI provide complementary information.

Given that there is no direct correspondence between DECOMPOSE‐QSM and NODDI parameters due to fundamentally different biophysical models, a visual comparison between χDCI, NDI and 1‐ISO was performed for the healthy cohort. NDI is a marker for neurite density, while 1‐ISO can be potentially interpreted as the intracellular volume fraction with ISO being the free water fraction. Average maps showed that NDI more closely resembles χDCI than 1‐ISO. This difference suggests that the C_0_ value from DECOMPOSE‐QSM and ISO from NODDI characterize different microstructural contents. In other words, the tissue volume accounted for by C_0_ does not fully overlap with structures characterized by isotropic diffusion, as ISO does. For example, extracellular space filled with fluid with restricted or hindered diffusion between parallel tight axons may be accounted for by C_0_, but not ISO.

Overall, the present findings support the use of χDCI as a new tool to provide information on tissue cellularity. Iron‐rich areas such as the deep gray nuclei as well as highly myelinated structures are well represented in χDCI. χDCI is proposed as a marker of cellularity rather than of cell bodies only; that is, it reflects the fraction of the volume (i.e., of a voxel) occupied by the cells. Specifically, it does not provide a measure of the number of cells. In gray matter regions, it is dominated by cell bodies, while in white matter areas it is a proxy of axonal density and glial cells. In the CSF χDCI values are close to zero, which agrees with the fact that CSF is composed mainly of fluid.

### 4.1 Similarities Between χDCI and NDI

The significant correlation observed across all the ROIs indicates an association between the measurements at the whole brain level. Though, because of the different sensitivities towards different cellular components, regional variations among the correlations between χDCI and NDI are expected. Positive correlations found in white matter are consistent with the fact that it is mainly composed of neurites. Both χDCI and NDI are in fact sensitive to myelinated axons and dendrites. NDI represents neurites density, while χDCI captures the contribution of myelin to the diamagnetic susceptibility compartment. As a result, NDI highlights white matter; χDCI on the other hand is a mixture of both neurites (C_−_) and cell bodies (C_+_), resulting in a different contrast between gray and white matter, the latter exhibiting a lower χDCI value.

The positive correlations observed in putamen, pallidum and thalamus can be explained by their anatomical composition, also characterized by neuronal projections and dendrites to which χDCI and NDI are both sensitive to. The acquisition protocols for diffusion data differ between the healthy and patient groups, which likely contributed to the variations between the groups. In fact, just based on the resolution, the signal‐to‐noise ratio (SNR) of the patient cohort data is half compared to that of the healthy cohort. As a result, the NDI of deep gray nuclei, such as the pallidum region, is artifactually high in the patient group. In the tumor regions, edema showed a positive correlation between χDCI and NDI, because, despite being composed mainly of fluid, it contains a relatively low number of cells or neurites [45].

### 4.2 Differences Between χDCI and NDI

Non‐significant correlations in some deep gray nuclei regions and cortical gray matter are expected to some extent given the differences in the biophysical model between NODDI and DECOMPOSE‐QSM. While the NODDI model was specifically developed for NDI to map neurites [11], DECOMPOSE‐QSM, thus χDCI, is applicable to all regions. In fact, in the deep gray nuclei most of the cellular contribution is given by glial cells, followed by neurons and their projections [46]. In the gray matter, NDI only reflects a small fraction of intracellular volumes due to the relatively low content of myelinated or non‐myelinated axons and dendrites, while χDCI also includes the contribution of paramagnetic sources such as cell bodies. Consequently, these findings suggest that χDCI might be more suitable for mapping tissue cellularity throughout the whole brain, without limiting its use to specific areas. For example, no significant correlation was found for necrosis and contrast enhancement areas as these regions are composed of a mixture of cell types and blood vessels rather than neurites mainly. Furthermore, the significant correlations and the negligible bias found between mean ROIs of the two populations suggest that χDCI is less dependent on the acquisition protocol and the MRI scanner, consequently providing higher reproducibility across different participant cohorts. Despite the significant bias observed for NDI in the Bland–Altman analysis, plots show that the estimated differences are within the limits of agreement and it is worth noting that the sample size for the analysis was small, potentially affecting the statistical significance. Furthermore, together with the strong correlation found, these results suggest that the NDI values are comparable between the two cohorts. Future studies comparing χDCI with the metrics of more advanced diffusion‐based techniques, such as SANDI, which models also cell bodies but requires much higher *b*‐values and longer acquisitions [47] are warranted.

### 4.3 Potential Utilities of χDCI

χDCI presents some advantages compared to other diffusion techniques. First, it relies on a faster acquisition sequence, i.e., the multi‐echo GRE sequence, which is already available on nearly all clinical scanners. Second, the spatial resolution that can be achieved for χDCI [48, 49] is higher than that for NDI due to the faster acquisition speed and lower sensitivity to motion of the GRE sequence. Third, χDCI is suitable for mapping tissue cellularity in neurological conditions of the brain. For example, tumor cell infiltration in peritumoral regions, such as the edema area, might be detected with χDCI. The T2‐FLAIR image is usually employed for the delineation of the edema region that appears as a homogeneous hyperintense area. In comparison, χDCI suggests a complex tissue composition within the edema region where NDI generally appears more homogeneous. Moreover, the combination of PCS, DCS and χDCI may be useful for the characterization of neurodegenerative diseases, in which excess iron deposition, microglia activation, demyelination and loss of cells coexist. For example, in Parkinson's disease it would be helpful to distinguish between the loss of neurons and the mere loss of their functioning [50], while multiple sclerosis patients would benefit from the differentiation between demyelination, cell loss and inflammatory cell accumulation [3]. Also, in the case of Alzheimer's disease it would be important to properly separate contributions of the multiple processes involved at once, such as iron deposition, protein accumulation and atrophy [1].

### 4.4 Limitations

Despite the promising results, the present work has some limitations. First of all, no ground truth or gold standard was available for comparison with the proposed method. Even though the NODDI model has been so far one of the most widely used, other, more advanced, diffusion models have been developed but comparing them with χDCI was outside the scope of the present work. Rician noise bias correction was not applied to diffusion data which may have caused spurious signal increase in low SNR areas, even though this is usually the case at stronger diffusion weighting and higher spatial resolution. Moreover, given the small sample size of the cohorts and the inclusion of only one type of pathology, studies including more subjects and different patient populations are warranted to assess the generalizability of the results. Nevertheless, first evidence of the reproducibility of the findings is supported by results observed using magnetic susceptibility maps computed with two dipole inversion methods. Specifically, the qualitative comparison of the two sets of DECOMPOSE‐QSM outputs provides visually comparable maps and the correlation results obtained with STAR‐QSM agree with the ones observed by using iLSQR. The bias found by the Bland–Altman analysis is very small (< 1%) compared to the χDCI range (i.e., [0,1]) and the measurements are randomly distributed within the limits of agreement. Ideally, χDCI measurements would benefit from histological validation to investigate how different aggregates of cells affect its value. In fact, given the highly complex tissue microstructure, there are some confounding effects that need to be considered when interpreting χDCI. Changes in the magnetic susceptibility sources (e.g., iron, lipid, and calcium) will indirectly affect χDCI. For example, not fully myelinated axons will exhibit a lower amplitude of magnetic susceptibility, resulting in an underestimation of χDCI. Similarly, very low iron concentration within the cell may result in inaccurate estimation of the volume fraction due to low sensitivity. Hemorrhages and calcifications will affect the magnetic susceptibility of the tissue resulting in an overestimation of χDCI. Extreme pathological conditions deviating from model assumptions are a common issue that affects each biophysical model.

### 4.5 Conclusion

χDCI is described to map tissue cellularity in healthy and lesioned areas of the brain. The significant correlations found with NDI as estimated from the NODDI model confirm the hypothesis that χDCI represents the intracellular volume fraction. More in general, χDCI may serve as a marker of tissue cellularity, a biologically and pathologically highly useful characterization of tissue microstructure. With relevance to tumor infiltration and monitoring of neurodegenerative diseases, χDCI can potentially support the clinical management of these pathological conditions.

## Funding

Supported in part by NIH through grants R01AG070826, R01MH127104 and by Weill Neurohub.

## Supporting information


**Table S1:** Correlation coefficients (*r*) and Benjamini‐Hochberg corrected *p*‐values (*p*) of χDCI versus NDI in the patient cohort. χDCI was estimated with magnetic susceptibility maps computed by using either iLSQR or STAR‐QSM dipole inversion algorithms. *indicates statistical significance.
**Figure S1:** Representative axial slices of group averaged maps computed in MNI space. The first row shows ISO, while the second row shows 1‐ISO.
**Figure S2:** Scatterplots of the associations between χDCI and NDI of the ROIs in the healthy (on the left) and the patients (on the right) cohorts. *r* indicates the correlation coefficient. Data points are reported as “.”, the fit is represented by the continuous line and the confidence bounds are indicated by the dotted lines.
**Figure S3:** Significant associations between χDCI and NDI in the investigated healthy ROIs for the patient cohort. Results here reported refer to χDCI estimated with STAR‐QSM‐derived susceptibility maps. *r* indicates the correlation coefficient. Data points are reported as “.”, the fit is represented by the continuous line and the confidence bounds are indicated by the dotted lines.
**Figure S4:** Bland–Altman plots for χDCI values derived from STAR‐QSM and iLSQR dipole inversion methods. The analysis was carried out for apparently healthy tissues (on the left) and the segmented tumor tissues (on the right) of the glioblastoma patients cohort. Continuous and dashed lines represent the mean difference and the limits of agreement, respectively. Error bars indicate the 95% confidence intervals.

## Data Availability

Data are available upon reasonable request and after the arrangement of a proper data sharing agreement as permitted by IRB.
