# Supplementary material for: Mapping Intracellular Volume Fraction With Susceptibility Source Decomposition as a Marker for Tissue Cellularity
Source: J Magn Reson Imaging. 2025 Dec 10;63(4):1092–105. doi: 10.1002/jmri.70194 (PMC12963808; doi:10.1002/jmri.70194)
Supplement: Supplementary file 1 — Table S1: Correlation coefficients (r) and Benjamini‐Hochberg corrected p‐values (p) of χDCI versus NDI in the patient cohort. χDCI was estimated with magnetic susceptibility maps computed by using either iLSQR or STAR‐QSM dipole inversion algorithms. *indicates statistical significance. Figure S1: Representative axial slices of group averaged maps computed in MNI space. The first row shows ISO, while the second row shows 1‐ISO. Figure S2: Scatterplots of the associations between χDCI and NDI of the ROIs in the healthy (on the left) and the patients (on the right) cohorts. r indicates the correlation coefficient. Data points are reported as “.”, the fit is represented by the continuous line and the confidence bounds are indicated by the dotted lines. Figure S3: Significant associations between χDCI and NDI in the investigated healthy ROIs for the patient cohort. Results here reported refer to χDCI estimated with STAR‐QSM‐derived susceptibility maps. r indicates the correlation coefficient. Data points are reported as “.”, the fit is represented by the continuous line and the confidence bounds are indicated by the dotted lines. Figure S4: Bland–Altman plots for χDCI values derived from STAR‐QSM and iLSQR dipole inversion methods. The analysis was carried out for apparently healthy tissues (on the left) and the segmented tumor tissues (on the right) of the glioblastoma patients cohort. Continuous and dashed lines represent the mean difference and the limits of agreement, respectively. Error bars indicate the 95% confidence intervals. [file JMRI-63-1092-s001.docx]

**Supplementary Information:**

| **ROI name** | **iLSQR** | **STAR-QSM** |
| --- | --- | --- |
| Cortical GM | *r* = 0.34 (*p* = 0.2) | *r* = 0.38 (*p* = 0.1) |
| WM | *r* = 0.6 (*p* = 0.01)* | *r* = 0.56 (*p* = 0.02)* |
| Thalamus | *r* = 0.64 (*p* = 0.007)* | *r* = 0.69 (*p* = 0.002)* |
| Caudate | *r* = 0.075 (*p* = 0.7) | *r* = 0.061 (*p* = 0.7) |
| Putamen | *r* = 0.79 (*p* = 1⋅10^-4^)* | *r* = 0.81 (*p* = 5⋅10^-5^)* |
| Pallidum | *r* = 0.48 (*p* = 0.05)* | *r* = 0.54 (*p* = 0.02)* |
| Hippocampus | *r* = 0.18 (*p* = 0.5) | *r* = 0.16 (*p* = 0.6) |
| Amygdala | *r* = -0.084 (*p* = 0.7) | *r* = 0.061 (*p* = 0.7) |
| Contrast enhancement | *r* = -0.07 (*p* = 0.7) | *r* = -0.068 (*p* = 0.7) |
| Necrosis | *r* = 0.29 (*p* = 0.2) | *r* = 0.36 (*p* = 0.1) |
| Edema | *r* = 0.69 (*p* = 0.001)* | *r* = 0.68 (*p* = 0.002)* |
| **Table S1.** Correlation coefficients (*r*) and Benjamini-Hochberg corrected p-values (*p*) of χDCI vs. NDI in the patient cohort. χDCI was estimated with magnetic susceptibility maps computed by using either iLSQR or STAR-QSM dipole inversion algorithms. *indicates statistical significance | | |

| **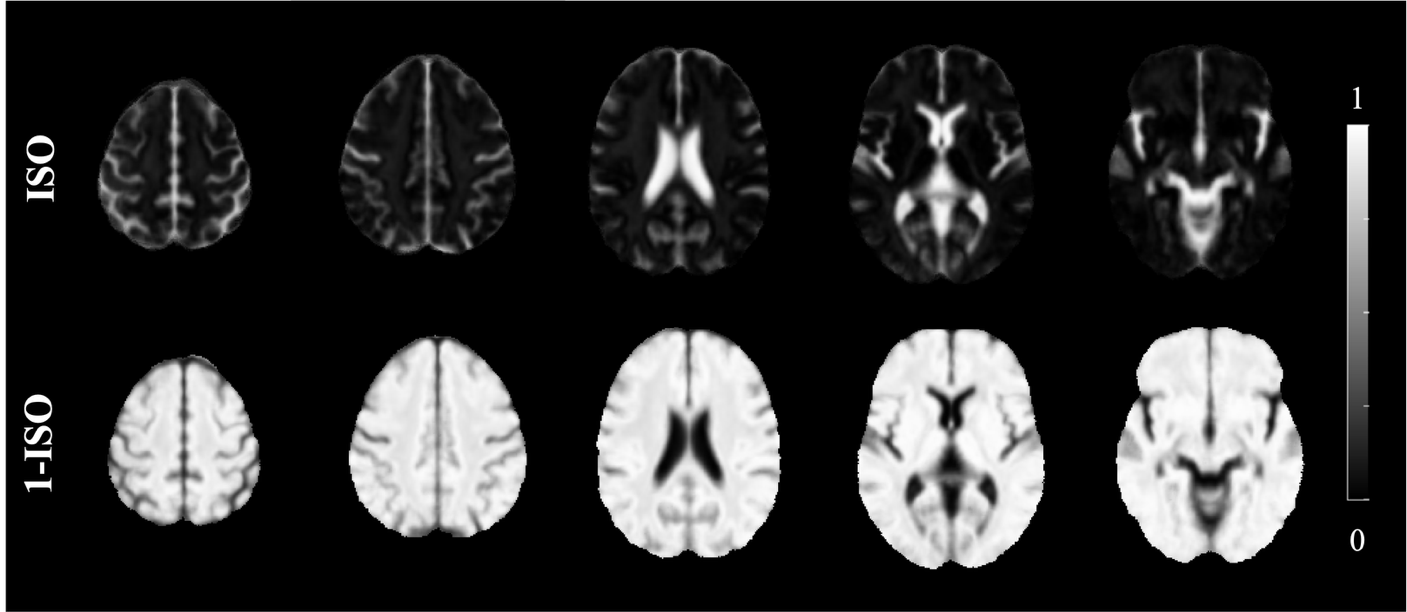** |
| --- |
| **Figure S1.** Representative axial slices of group averaged maps computed in MNI space. The first row shows ISO, while the second row shows 1-ISO. |

| 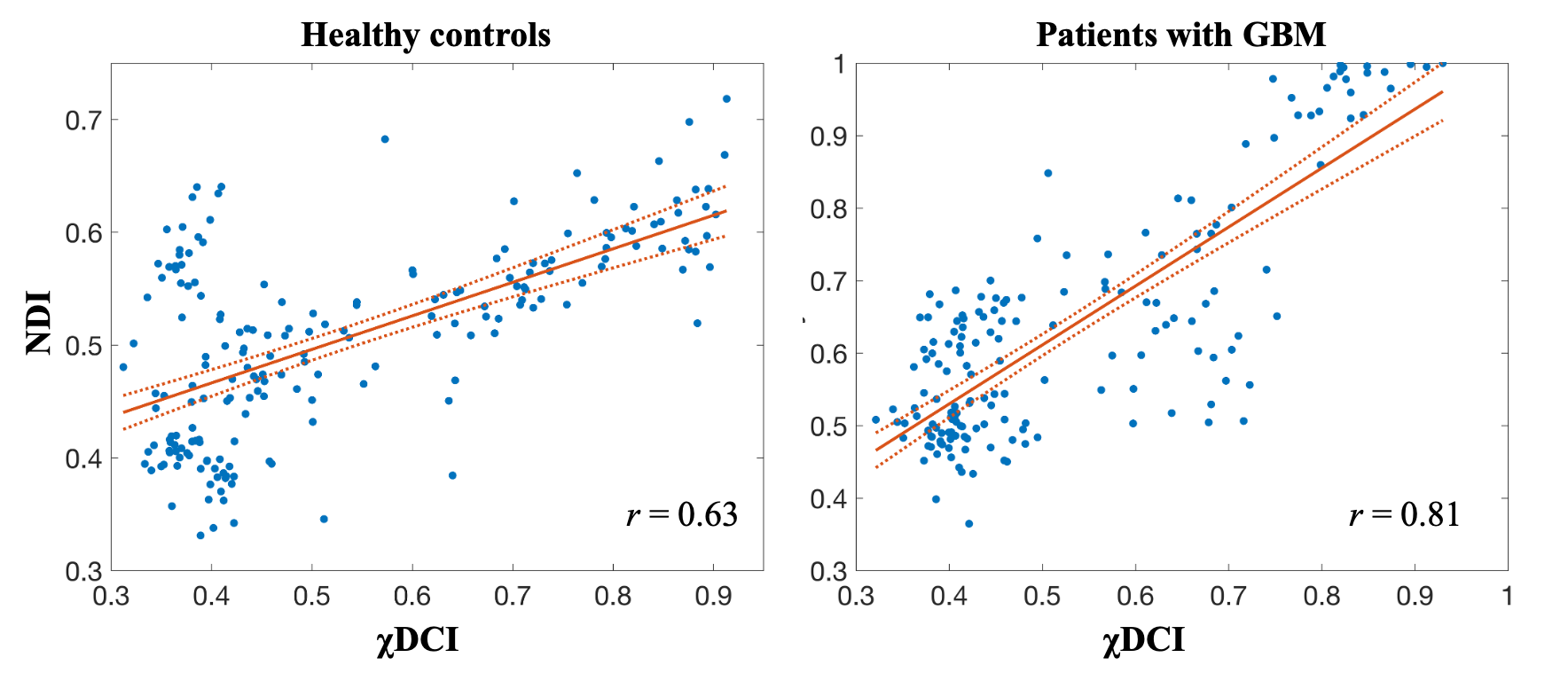 |
| --- |
| **Figure S2.** Scatterplots of the associations between χDCI and NDI of the ROIs in the healthy (on the left) and the patients (on the right) cohorts. r indicates the correlation coefficient. Data points are reported as “.”, the fit is represented by the continuous line and the confidence bounds are indicated by the dotted lines. |

| 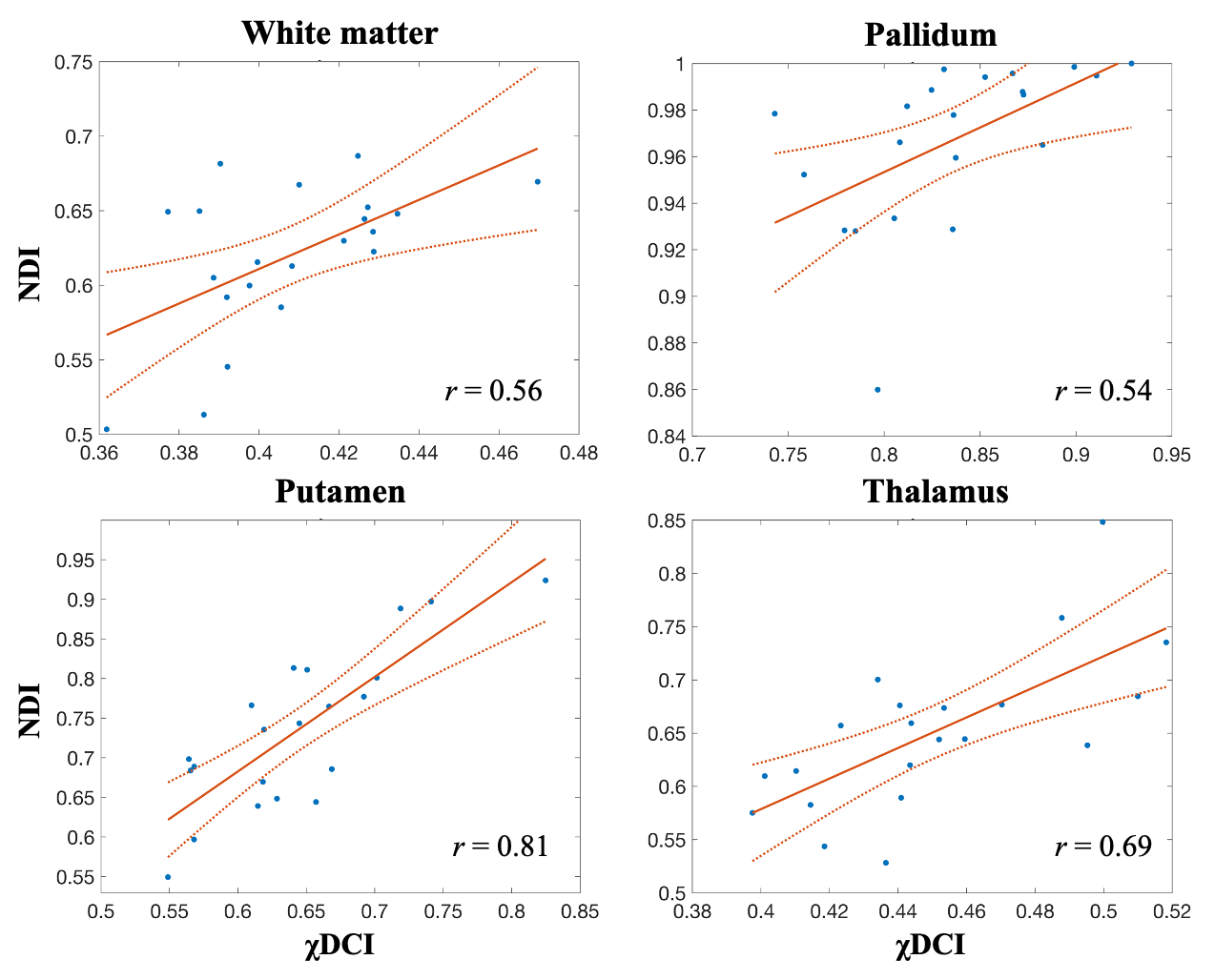 |
| --- |
| **Figure S3.** Significant associations between χDCI and NDI in the investigated healthy ROIs for the patient cohort. Results here reported refer to χDCI estimated with STAR-QSM-derived susceptibility maps. r indicates the correlation coefficient. Data points are reported as “.”, the fit is represented by the continuous line and the confidence bounds are indicated by the dotted lines. |

| 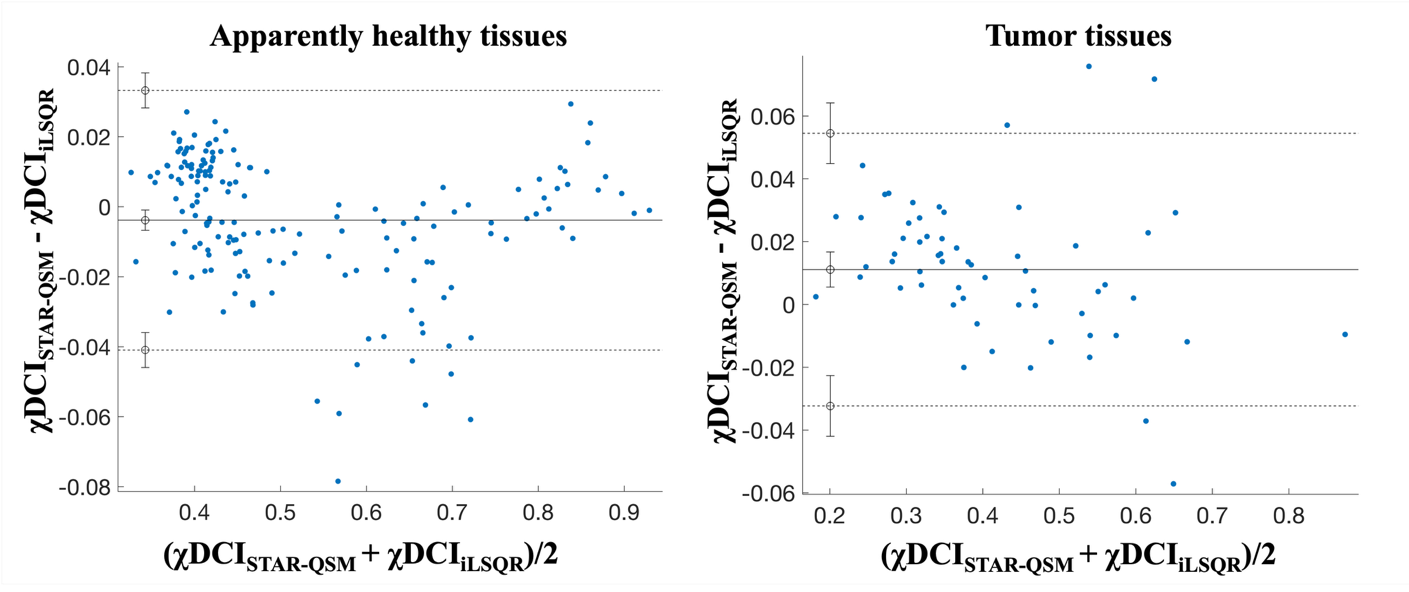 |
| --- |
| **Figure S4.** Bland-Altman plots for χDCI values derived from STAR-QSM and iLSQR dipole inversion methods. The analysis was carried out for apparently healthy tissues (on the left) and the segmented tumor tissues (on the right) of the glioblastoma patients cohort. Continuous and dashed lines represent the mean difference and the limits of agreement, respectively. Error bars indicate the 95% confidence intervals. |
